# The Acute Effects of External Compression With Blood Flow Restriction on Maximal Strength and Strength-Endurance Performance of the Upper Limbs

**DOI:** 10.3389/fphys.2020.00567

**Published:** 2020-06-10

**Authors:** Michal Wilk, Michal Krzysztofik, Aleksandra Filip, Robert G. Lockie, Adam Zajac

**Affiliations:** ^1^Institute of Sport Sciences, Jerzy Kukuczka Academy of Physical Education in Katowice, Katowice, Poland; ^2^Center for Sports Performance, Department of Kinesiology, California State University, Fullerton, Fullerton, CA, United States

**Keywords:** occlusion, bench press, pressure, 1RM test, time under tension, repetitions

## Abstract

The main goal of the present study was to evaluate the acute effects external compression with blood flow restriction (BFR) at 100 and 150% of full arterial occlusion pressure (AOP) on maximal strength and strength-endurance performance during the bench press (BP) exercise. The study included 12 strength-trained male subjects (age = 23.2 ± 2.66 years; body mass = 75.3 ± 6.33 kg; height = 179.1 ± 3.82 cm), experienced in resistance training (5.7 ± 2.93 years). During the experimental sessions in a randomized crossover design, the subjects performed a 1 repetition maximum (1RM) test and three sets of the BP using 60% 1RM to failure with three different conditions: without BFR (NO-BFR); BFR with a pressure of 100% AOP (BFR_100_); and BFR with a pressure of 150% AOP (BFR_150_). The differences between the NO-BFR, BFR_100_, and BFR_150_ conditions were examined using repeated measures ANOVA. The ANOVA indicated significant main effect for condition in 1RM, number of performed repetitions (REP), and time under tension (TUT) (*p* < 0.01). *Post hoc* analyses for the main effect indicated significant increases in 1RM (*p* < 0.01; 95.00 ± 15.37 vs 91.87 ± 15.99), REP (*p* < 0.01; 17.56 ± 3.36 vs 15.67 ± 5.24), and TUT (*p* < 0.01; 32.89 ± 6.40 vs 28.72 ± 6.18) for the BFR_150_ condition compared to NO-BFR. Furthermore, significant increases in REP (*p* = 0.03; 17.56 ± 3.36 vs 16.47 ± 4.01) and TUT (*p* = 0.03; 32.89 ± 6.40 vs 30.00 ± 6.45) were observed for the BFR_150_ condition compared to the BFR_100_. The results of the present study indicate that high external compression increases maximal strength evaluated by the 1RM test, as well as endurance performance during three sets of the BP exercise.

## Introduction

Resistance training is a primary exercise intervention used to develop strength and stimulate muscle hypertrophy. Besides the basic methods of resistance training, research has focused on the use of additional training equipment or devices that may enhance performance and the resulting training adaptations. One of these training methods includes blood flow restriction (BFR). BFR during resistance exercise has been used in physical therapy and in the training process of both physically active people and competitive athletes, with the objective to increase muscle hypertrophy and strength (Takarada et al., 2000; Cook et al., 2014). The BFR technique involves the use of a tourniquet, an inflatable cuff, or elastic wraps that exert high pressure at the proximal part of the limb, which through external compression reduces arterial blood flow and shuts venous blood flow during physical exercise (Loenneke and Pujol, 2009; Wilk et al., 2018b). Due to the variety of widths, length, shape, and material of the cuff, as well as individual limb characteristics (Loenneke et al., 2012b, 2015), it is recommended to set the pressure according to the individuals value of arterial occlusion pressure (% AOP) (Patterson et al., 2019).

Despite the fact that much attention has been devoted to the use of BFR during resistance exercise, most of the focus has been on chronic adaptive hypertrophy changes in subjects with a low level of experience in resistance exercise (Fujita et al., 2007; Madarame et al., 2008; Manimmanakorn et al., 2013; Wilk et al., 2018b). Only few studies have compared the acute effects of BFR on strength-endurance performance (Wernbom et al., 2006, 2009; Loenneke et al., 2012a) and only one study has examined its effects in multi-joint movements of the upper limbs (Rawska et al., 2019). Wernbom et al. (2009) and Loenneke et al. (2012a) found that significantly lower repetitions (REP) were performed during the leg extension under BFR compared to conditions without BFR (NO-BFR) at 30% 1 repetition maximum (1RM). This result was partly compatible with a previous study by Wernbom et al. (2006) who also showed a significantly lower number of REP performed under BFR compared to NO-BFR with loads of 20, 30, and 40% 1RM. However, there was no difference at higher loads (50% 1RM). Thus, the application of a pressure cuff around the thigh appears to reduce knee extension endurance performance more at a low load than at a moderate one. However, these data were contrary to the study by Rawska et al. (2019) which showed an increase in the number of performed REP (five sets) during the bench press (BP) exercise at a load of 80% 1RM under BFR compared to NO-BFR conditions. It should be noted that the differences in results obtained in the studies by Wernbom et al. (2006, 2009) and Rawska et al. (2019) can be related to both the external load used, type of movement (single-joint vs multi-joint), the occluded muscle area (upper limb, lower limb), and most notably the cuff width and cuff pressure used. Currently, there is no standard cuff width and pressure for BFR training, with the cuff width used in previous studies ranging from 5 to 14 cm (Fujita et al., 2007). Loenneke et al. (2012b) demonstrated that a constant pressure of occlusion with a wide cuff resulted in different physiological and adaptive responses compared to a narrow cuff (Loenneke et al., 2012b; Rossow et al., 2012), which could be one of the main reasons for the significant differences in results of the aforementioned studies (Wernbom et al., 2006, 2009; Rawska et al., 2019).

The primary mechanisms responsible for the adaptive responses associated with training under BFR conditions include increased elevated metabolic stress and mechanical tension compared to traditional resistance training (Pearson and Hussain, 2015; Teixeira et al., 2018). However, Rawska et al. (2019) suggested that mechanical work generated by the cuff can be an additional factor that may influence the effectiveness of resistance training under BFR, especially when high external compression is used. A cuff is a passive element, but during the movement, especially in the eccentric contraction, the strain of the material of which the cuff is made may produce additional elastic energy what can increase performance when recoiling (Rawska et al., 2019; Wilk et al., 2020c). Such an increase in performance through external compression is similar to that observed in competitive powerlifting, where compressive gear is used (squat suits, BP shirts, deadlift suits) (Doan et al., 2003; Godawa et al., 2012). Confirmation of the theory that the mechanical work of the cuff is related with the external compression can be a significant factor affecting the level of performance as shown recently by Wilk et al. (2020c). Wilk et al. (2020c) indicated that short-term, high pressure occlusion increases power output and bar velocity during the BP exercise. However, the authors of the study indicated that these results may not translate to other types of exercises, with different external loads, what requires further research.

Due to the lack of scientific data concerning the acute effects of BFR during resistance exercise on strength performance, the aim of the present study was to evaluate the effects of external muscle compression with full BFR on maximal strength and strength-endurance performance during the BP exercise. An additional goal of the present study was to assess the impact of external compression and the associated mechanical work generated by the cuff on level of strength performance (without changing the level of BFR). Therefore, the authors decided to use two values of pressure, both causing complete shutting down of blood flow (100 and 150% AOP). It was hypothesized that a higher pressure cuff would be more effective in inducing an increase in performance compared to a lower pressure cuff.

## Materials and Methods

The experiment followed a randomized crossover design, where each subject performed three familiarization sessions, one session of 1RM testing, and three different testing conditions: without BFR (NO-BFR); BFR with pressure of 100% AOP (BFR_100_); and BFR with a pressure of 150% AOP (BFR_150_). The research procedure lasted 7 weeks with a 1 week interval between each trial. During the experimental sessions, subjects performed the 1RM test, and the strength-endurance test at 60% 1RM to momentary concentric failure.

### Subjects

Twelve healthy strength-trained men (age = 23.2 ± 2.66 years; body mass = 75.3 ± 6.33 kg; height = 179.1 ± 3.82 cm), experienced in resistance training (5.7 ± 2.93 years) volunteered for the study after completing an informed consent form. The inclusion criterion was a BP personal record of at least 120% of body mass. The subjects were allowed to withdraw from the experiment at any time and were free from musculoskeletal disorders. The subjects were instructed to maintain their normal dietary habits over the course of the study and not to use any supplements or stimulants for the duration of the experiment. All subjects were informed about the benefits and potential risks of the study before providing their written informed consent. The study subjects were required to refrain from resistance training 72 h before each experimental session. The experimental protocol was approved by the Bioethics Committee for Scientific Research, at the Academy of Physical Education in Katowice, Poland (2/2019) and conducted according to the ethical standards of the Declaration of Helsinki, 1983.

### Familiarization Session and 1RM Strength Test

Four weeks before the main experiment, the athletes performed several familiarization sessions once per week. During the familiarization sessions, the athletes performed three sets of three repetitions of the BP with a load of perceived 80% 1RM. Two sets were performed under BFR_100_ and two sets under BFR_150_. The familiarization sessions were performed in order to restrict possible learning effects. A familiarization session preceded the preliminary 1RM testing. Subjects arrived at the laboratory at the same time of day as in the upcoming experimental sessions (between 9:00 and 11:00 am). Upon arrival, the subjects cycled on an ergometer for 5 min at an intensity that resulted in a heart rate of approximately 130 bpm, followed by a general upper body warm-up. Next, they performed 15, 10, 5, and 3 repetitions of the BP using 20, 40, 60, and 80% of their estimated 1RM with a 2/0/X/0 tempo of movement. The sequence of digits describing the tempo of movement (2/0/X/0) referred to a 2 s eccentric phase, 0 represented a pause during the transition phase, X referred to the maximum possible tempo of movement during the concentric phase, and the last digit indicated no pause at the end of movement (Wilk et al., 2019). Subjects then executed single repetitions of the BP with a 5-min rest interval between successful trials. The load for each subsequent attempt was increased by 2.5–10 kg, and the process was repeated until failure. Hand placement on the barbell was individually selected which represented a grip width on the barbell of ∼150% individual bi-acromial distance. No BP suits, weightlifting belts, or other supportive garments were permitted. Three spotters were present during all attempts to ensure safety and technical proficiency.

### Experimental Sessions

Three testing sessions were used for the experimental trials and the protocols were identical. All testing took place between 9.00 and 11.00 am to avoid circadian variation. The general warm-up for the experimental sessions was identical to the one used for the familiarization session. After warming-up, the subjects performed the 1RM BP test to assess upper-body maximal strength. The 1RM test is considered the gold standard for assessing muscle strength under non-laboratory conditions (Levinger et al., 2009). For the 1RM test, the first warm-up set included eight to ten repetitions with 50% 1RM determined during the familiarization session. The second set included three to five repetitions with 75% 1RM. The subjects then completed one repetition with 95% 1RM with a constant movement tempo 2/0/X/0 (Wilk et al., 2020a,b). Based on whether the subject successfully lifted the load or not, the weight was increased or decreased (2.5–10 kg) in subsequent attempts until the 1RM value was reached. Five minute rest intervals were allowed between successive 1RM attempts, and all 1RM values were obtained within five attempts. After a 5 min rest interval, strength-endurance was assessed with three “all-out” sets using a load of 60% 1RM measured in the previous test. Five minute rest intervals were allowed between sets. The strength-endurance test was terminated when momentary concentric failure occurred. The concentric and eccentric phase was performed at maximal possible velocity in each repetition.

All repetitions were performed without bouncing the barbell off the chest, without intentionally pausing at the transition between the eccentric and concentric phases, and without raising the lower back off the bench. A linear position transducer system (Tendo Power Analyzer, Tendo Sport Machines, Trencin, Slovakia) was used for the evaluation of bar velocity. The Tendo Power Analyzer is a reliable system for measuring movement velocity and to estimate power output (Goldsmith et al., 2019). The measurement was made independently in each repetition and automatically converted into the values of power (peak, mean) and bar velocity (peak, mean). During the experimental sessions, the following variables were registered:

1. 1RM—1 repetition maximum (kg).
2. REP—number of repetitions in a set (n).
3. TUT—time under tension in each set (s).
4. PP—peak power output (W).
5. MP—mean power output (W).
6. PV—peak velocity (m/s).
7. MV—mean velocity (m/s).

### Blood Flow Restriction

During exercise Smart Cuffs were applied (Smart Tools Plus LLC, Strongsville, OH, United States). The subjects wore cuffs at the most proximal region of both arms during the experimental sessions (Takarada et al., 2002). In order to determine the individual occlusion pressure, the value of full AOP at rest was determined. The measurement was conducted twice on each limb and the obtained differences were within 20 mmHg, the average was then used to set the cuff pressure for exercise. Cuff pressure for exercise was set to the value of 100 or 150% full AOP (135 mmHg ± 16 for BFR_100_; 202 mmHg ± 23 for BFR_150_). The level of vascular restriction was controlled by a hand-held Edan SD3 Doppler with an OLED screen and a 2 mHz probe made by Edan Instruments (Shenzen, China). The restriction of muscular blood flow was applied immediately before the start of the exercise set and released immediately upon completion of the last repetition. During the rest interval, the occlusion was not applied (Wilk et al., 2020c).

### Statistical Analysis

All statistical analyses were performed using Statistica 9.1. Results are presented as means with standard deviations. The Shapiro–Wilk, Levene, and Mauchly’s tests were used in order to verify the normality, homogeneity, and sphericity of the sample data variances, respectively. Any differences between the NO-BFR, BFR_100_, BFR_150_ conditions for the 1RM test were examined using repeated measures one-way ANOVA, while the strength-endurance test were examined using repeated measures two-way ANOVA 3 × 3 (conditions × set). The statistical significance was set at *p* < 0.05. Whenever a significant main effect occurred, *post hoc* comparisons were conducted using the Tukey’s test. Percent changes and 95% confidence intervals were also calculated. Effect sizes (Cohen’s *d*) were reported where appropriate. Parametric effect sizes were defined as: large (*d* > 0.8); moderate (*d* between 0.79 and 0.5); small (*d* between 0.49 and 0.20) and trivial (*d* < 0.2) (Cohen, 1988).

## Results

**FIGURE 1:**
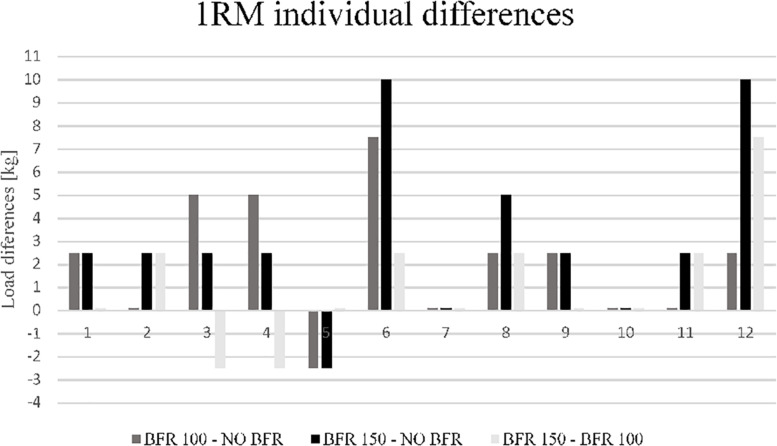
Individual responses for the IRM test between conditions.

The two-way repeated measures ANOVA indicated significant condition × set interaction effect for REP (*p* < 0.01). There was a significant main effect for condition in REP and TUT (*p* < 0.01) (Table 1). The one-way repeated measures ANOVA indicated significant condition interaction in 1RM test results (*p* < 0.01; Table 1 and Figure 1).

**TABLE 1:** Main conditions effect in the 1RM test and during three sets of the bench press under the three employed conditions.

Bench press	Conditions			
	NO-BFR	BFR_100_	BFR_150_	*P*
1RM [kg]	91.87 ± 15.99	93.95 ± 15.94	95.00 ± 15.37	0.01*
Number of repetitions [n]	15.67 ± 5.24	16.47 ± 4.01	17.56 ± 3.36	0.01*
Time under tension [s]	28.72 ± 6.18	30.00 ± 6.45	32.89 ± 6.40	0.01*
Peak power output [W]	580 ± 131	575 ± 118	584 ± 114	0.94
Mean power output [W]	262 ± 56	275 ± 41	273 ± 44	0.39
Peak velocity [m/s]	0.89 ± 0.18	0.89 ± 0.17	0.90 ± 0.15	0.92
Mean velocity [m/s]	0.48 ± 0.09	0.50 ± 0.09	0.49 ± 0.08	0.54

All data are presented as mean ± standard deviation. *Statistical significance *p* < 0.05.

*Post hoc* analyses for main effect indicated significant increases in 1RM, REP, and TUT during BFR_150_ compared to NO-BFR (*p* < 0.01), as well significant increases in REP and TUT during BFR_150_ compared to BFR_100_ (*p* = 0.03). *Post hoc* analyses for REP (condition × set) showed a significant increase in REP_*SET*__2_ in BFR_150_ compared to NO-BFR, and a significant increase in REP_*SET*__3_ in BFR_100_ compared to NO-BFR, as well as a significant increase in REP_*SET*__3_ in BFR_150_ compared to NO-BFR (Table 2). There were no significant differences in PP, MP, PV, MV between NO-BFR, BFR_100_, and BFR_150_ (Supplementary Data Sheet S1).

**TABLE 2:** Differences in particular conditions between the 1RM test and the three experimental bench press sets.

Bench press		Conditions			Effect size Cohen *d*		
		NO-BFR (95% CI)	BFR_100_ (95% CI)	BFR_150_ (95% CI)	NO-BFR vs BFR_100_	NO-BFR vs BFR_150_	BFR_100_ vs BFR_150_
1RM [kg]		91.87 ± 15.99* (81.71 to 102.04)	93.95 ± 15.94 (83.83 to 104.08)	95.00 ± 15.37* (85.23 to 104.77)	0.13	0.19	0.07
REP [n]	Set 1	22.08 ± 1.92 (20.85 to 23.30)	20.83 ± 1.90 (19.63 to 22.04)	20.67 ± 2.64 (18.99 to 22.34)	0.65	0.61	0.06
REP [n]	Set 2	14.66 ± 1.67* (13.60 to 15.73)	16.33 ± 1.51 (15.38 to 17.28)	17.91 ± 1.31* (17.08 to 18.75)	1.04	2.16	1.12
REP [n]	Set 3	10.25 ± 1.66* (9.20 to 11.30)	12.25 ± 2.26* (10.81 to 13.69)	14.08 ± 1.83* (12.92 to 15.25)	1.01	2.19	0.89
TUT [s]	Set 1	34.42 ± 4.17 (31.76 to 37.06)	35.67 ± 4.56 (32.74 to 38.59)	36.68 ± 6.21 (32.72 to 40.61)	0.28	0.4	0.18
TUT [s]	Set 2	28.58 ± 3.87* (26.12 to 31.04)	29.33 ± 4.66 (26.37 to 32.29)	33.17 ± 5.59* (29.61 to 36.71)	0.17	0.95	0.74
TUT [s]	Set 3	23.16 ± 4.49* (20.31 to 26.09)	25.00 ± 5.15 (21.73 to 28.27)	28.83 ± 5.17* (25.55 to 32.12)	0.38	1.17	0.74
PP [W]	Set 1	653 ± 112 (582 to 725)	634 ± 114 (561 to 707)	628 ± 122 (550 to 706)	0.17	0.21	0.05
PP [W]	Set 2	574 ± 138 (487 to 662)	585 ± 117 (510 to 659)	598 ± 111 (527 to 669)	0.08	0.19	0.11
PP [W]	Set 3	510 ± 110 (439 to 580)	506 ± 91 (448 to 564)	524 ± 88 (467 to 580)	0.04	0.14	0.20
MP [W]	Set 1	309 ± 52 (275 to 343)	302 ± 39 (277 to 326)	288 ± 54 (254 to 323)	0.15	0.39	0.29
MP [W]	Set 2	251 ± 44 (222 to 279)	277 ± 33 (256 to 297)	274 ± 38 (250 to 299)	0.67	0.56	0.08
MP [W]	Set 3	227 ± 37 (203 to 251)	245 ± 32 (225 to 266)	254 ± 32 (233 to 274)	0.52	0.78	0.28
PV [m/s]	Set 1	1.04 ± 0.17 (0.93 to 1.15)	0.99 ± 0.15 (0.90 to 1.10)	0.97 ± 0.15 (0.87 to 1.06)	0.31	0.44	0.13
PV [m/s]	Set 2	0.87 ± 0.15 (0.78 to 0.97)	0.89 ± 0.14 (0.81 to 0.99)	0.92 ± 0.15 (0.82 to 1.02)	0.14	0.33	0.21
PV [m/s]	Set 3	0.76 ± 0.09 (0.71 to 0.83)	0.80 ± 0.16 (0.70 to 0.90)	0.82 ± 0.13 (0.74 to 0.91)	0.31	0.54	0.14
MV [m/s]	Set 1	0.56 ± 0.08 (0.51 to 0.62)	0.55 ± 0.08 (0.50 to 0.60)	0.52 ± 0.08 (0.46 to 0.57)	0.13	0.5	0.38
MV [m/s]	Set 2	0.46 ± 0.07 (0.42 to 0.50)	0.51 ± 0.08 (0.46 to 0.56)	0.50 ± 0.06 (0.46 to 0.54)	0.67	0.61	0.14
MV [m/s]	Set 3	0.42 ± 0.05 (0.38 to 0.45)	0.45 ± 0.08 (0.40 to 0.50)	0.46 ± 0.06 (0.41 to 0.50)	0.45	0.72	0.14

All data are presented as mean ± standard deviation; CI: confidence interval. *Statistical significance *p* < 0.05.

## Discussion

The main finding of the study was that external compression significantly increased results of the 1RM test, as well as the number of performed REP and TUT during the BP. Furthermore, the results also indicated that 1RM, REP, and TUT were significantly higher during the BP under BFR_150_ compared to the BFR_100_ conditions. This suggests that not only the use of BFR could be effective, but also the mechanical compression and related work generated by the cuff can be a potential factor affecting the acute increase in maximal strength and strength-endurance performance.

The current research analyzed two important aspects related to the effects of resistance exercise under BFR. The first is related with maximal strength, while the second is connected to strength-endurance. However, currently there is no available scientific data regarding the impact of short-term external compression on acute changes in maximal load during the 1RM test, which limits the possibility of comparing our results to other studies. Nevertheless, important information can be derived from the current study.

The results of the present study show significant increases in 1RM during BFR_150_ compared to NO-BFR, simultaneously showing no such effect between BFR_100_ compared to NO-BFR. The differences between BFR_100_ and BFR_150_ occurred despite the fact that both conditions (BFR_100_ and BFR_150_) caused full arterial occlusion, therefore the physiological level of metabolic stress should be similar for both conditions, which suggests that other than physiological factors had a significant effect on the obtained results. It is important to note that the occlusion applied during the 1RM test lasted only a few seconds. The short duration of the effort during the 1RM test would be fueled mainly by phosphocreatine and anaerobic glycolysis (Bogdanis et al., 1998), with relatively low levels of metabolic stress and fatigue. Based on this, it may be argued that in the current study metabolic stress was not very high, due to the short duration of effort. Therefore, the effectiveness of BFR was possibly less related to metabolic factors and more to mechanical factors, such as the mechanical energy accumulated in the cuff proportional to its width. Furthermore, confirmation that the mechanical energy accumulated in the cuff is the main factor causing improved 1RM performance is the fact that such an increase was observed only under pressure of 150% AOP. BFR with a pressure of 100% AOP did not cause changes in the 1RM result when compared to NO-BFR. This confirms previous data which suggests that cuff pressure affects the effectiveness of resistance exercise under BFR (Loenneke et al., 2012b; Rossow et al., 2012; Wilk et al., 2018b); however, the presented study is the first that used compression pressure above 100% AOP.

In addition to significant changes in the 1RM test, the current study also showed a significant increase in REP and TUT during BFR_150_ compared to NO-BFR and BFR_100_. The increase in the number of performed REP during BFR_150_ compared to NO-BFR is contrary to the results of Wernbom et al. (2009) and Loenneke et al. (2012a). Both of these studies showed a significantly lower number of REP during the leg extension at 30% 1RM under BFR compared to conditions without BFR. However, differences in both the occlusion pressure and the occluded muscle area may be the cause of divergent results. Only one previous study analyzed the impact of BFR on the number of performed REP in the multi-joint upper body exercise. Rawska et al. (2019) showed an increase in the number of REP during the BP exercise at 80% 1RM under BFR (80% AOP) compared to conditions without BFR. The result of our study is consistent with that of Rawska et al. (2019); however, our study also showed a significant increase in the number of REP during BFR_150_ compared to BFR_100_ conditions. The present study is the first which considered the changes in TUT under BFR. According to the guidelines of Wilk et al. (2018a), the value of TUT may be an accurate and credible indicator of work performed during a resistance training session. The present study showed significant differences in TUT when the BP was performed under BFR_150_ compared to BFR_100_ and NO-BFR. Although all three sets of the BP were performed to muscle failure, there was no decrease in exercise capacity associated with BFR (Wernbom et al., 2009; Loenneke et al., 2012a). On the contrary, there was an improvement in the number of performed REP and TUT during the BFR_150_ compared to NO-BFR and BFR_100_ conditions.

Likewise, in case of the analysis of the 1RM test results, the mechanical compression and related work generated by the cuff is a potential cause and explanation of the obtained increase in TUT and REP for the BFR_150_ condition. Research by Harman and Frykman (1990) confirmed that wearing knee wraps allows athletes to lift greater loads or perform more repetitions during a particular set. This phenomenon can be explained by the elastic energy generated as the knee wraps stretch during the lowering phase, and then returning the mechanical energy during the lifting phase (Harman and Frykman, 1990). A similar effect may apply to the occlusion cuffs. However, the occlusion cuff was not set up in the joint area, but on the muscle belly of upper limbs, thus this comparison may not be equivalent. A cuff is a passive element, but during movement (especially in the eccentric contraction), the strain of the material of which the cuff is made may provide additional elastic energy (Wilk et al., 2020c). As a result, energy released from the cuff during the concentric contraction could affect the 1RM, REP, and TUT increase during BFR_150_ when compared to NO-BFR or to BFR_100_. This effect may be similar to the one observed during powerlifting competition, where compressive gear is used (Inzer, Titan) which assists the athlete during the eccentric phase of the lift, giving a “rebound” effect during the concentric phase of the lift. The hypothesis that the mechanical energy accumulated in the cuff and not physiological changes is a contributing factor for the increase in REP and TUT is reinforced by the differences between BFR_150_ and BFR_100_ conditions. Cuff pressure for BFR_150_ and BFR_100_ was set to full vascular restriction. Although the physiological reactions were similar in both BFR_150_ and BFR_100_, the REP and TUT were significantly greater in BP under BFR_150_ when compared to BFR_100_. Significant differences between BFR_150_ and BFR_100_ may indicate that the pressure above 100%AOP can be an important factor determining acute changes during resistance exercise under BFR, due to its ability to store and recoil larger amounts of elastic energy during the BP. Furthermore, it can be assumed that the use of another exercise could cause different, even conflicting results. The prime movers involved in the BP exercise are the pectoralis major, anterior deltoid, and triceps brachii muscle (Gołaś et al., 2018); however, the external compression was used at the upper limb (arms), and this area does not affect the changes taking place in the pectoralis major and deltoid muscles. Therefore, the BFR was not sufficient to occlude blood flow to all prime movers during the lift (Godawa et al., 2012).

This study confirmed previous data which suggests that cuff pressure affects the effectiveness of resistance exercise under BFR (Wilk et al., 2018b); however, this study is the first that used compression pressure above 100% AOP. Therefore, the results are inconsistent with the statement of Loenneke et al. (2014) who suggested that excessive pressure of BFR does not lead to higher benefits compared to exercise at an optimal BFR pressure. However, the statement of Loenneke et al. (2014) was related to chronic changes, while the results of our study relate to acute responses.

The present study has several limitations which should be addressed. Although the results showed significant changes in 1RM test as well as in REP and TUT during the sets performed to muscular failure, the causes of these changes cannot be directly determined and explained. There was no direct analysis of physiological variables which could explain the obtained results. Furthermore the use of high pressure mechanical compression caused high discomfort as well skin injuries reported by subjects, especially during BFR_150_ condition. It should also be noted that long-term resistance training programs under BFR applied in one muscle area can impair the muscle structure directly in the region under the cuff, which is a huge risk not only for athletic performance, but also for the health and safety of the athlete (Kacin and Strazar, 2011; Ellefsen et al., 2015). Therefore, resistance training with mechanical compression cannot be used as a constant, frequent training tool.

### Practical Implications

The mechanical compression increases the level of strength as well as strength-endurance performance during the BP exercise. However, only high pressure of compression causes significant improvements in performance. Therefore, such a training tool can be used as an additional factor to help athletes and coaches in programming varied resistance training protocols. The increase in the level of maximal load under the mechanical compression can also be used as a form of training overload. However, it must be stated that the mechanical compression should not be used in every training session or even in every set of an exercise. It can be suggested that combining sets with, and without mechanical compression could provide optimal strength gains and reduce the risks related with extremely high mechanical muscle compression. Taking into account the training methodology of powerlifters (who commonly use compression gear) as well as unpublished research results from the Strength and Power Laboratory of the Academy of Physical Education in Katowice, high mechanical muscle compression should be used in two different training protocols. In the first training option, the sets with mechanical compression are combined with sets without compression (Table 3). The second option involves changes in the microcycle and thus the performance of one training unit with mechanical compression and another without mechanical compression (Table 4). Furthermore, it must be remembered that there are individuals who do not benefit from exercise under mechanical compression (Figure 1) which indicates the need for individualization in case of high pressure external muscle compression.

**TABLE 3:** A sample training program of the Polish, World, and European champion in the bench press (M.W.).

Order	Number of sets	%1RM	Number of repetitions	Mechanical compression
1	2	80%	4	NO
2	1	85%	3	NO
3	3	90%	3	NO
4	2	95%	2	YES
5	1	100%	2	YES
6	1	105%	1	YES
7	2	85%	3	NO

**TABLE 4:** A sample training program of the Polish, World, and European champion in powerlifting (M.W.).

Monday	Tuesday	Wednesday	Thursday	Friday	Saturday	Sunday
Barbell squat (compression)	Free day	Bench press (no-compression)	Deadlift (no-compression)	Bench press (compression)	Barbell squat (no-compression)	Bench press (no-compression)

*The example does not contain information about complementary exercises.*

## Conclusion

Short-term, high pressure BFR increases maximal strength in the 1RM test, and allows for a greater number of performed repetitions and longer TUT during several sets of an upper body multi-joint exercise performed to muscular failure. This suggests that high pressure BFR could be an important tool in eliciting greater strength and strength-endurance performance. This would allow for additional possibilities in periodization of resistance training programs. However, these results cannot apply to all athletes, and cannot be translated into other types of exercises. Therefore, there is a need for further research with resistance exercise under BFR.

## Data Availability Statement

The raw data supporting the conclusions of this article will be made available by the authors, without undue reservation, to any qualified researcher.

## Ethics Statement

The study protocol was approved by the Bioethics Committee for Scientific Research, at the Academy of Physical Education in Katowice, Poland (2/2019). The patients/participants provided their written informed consent to participate in this study.

## Author Contributions

MW and MK contributed to study conception and design and acquisition of data. MW, MK, and AF contributed to analysis and interpretation of data and drafting of the manuscript. MW, AZ, and RL contributed to critical revision.

## Conflict of Interest

The authors declare that the research was conducted in the absence of any commercial or financial relationships that could be construed as a potential conflict of interest.

## Supplementary Material

DATA SHEET S1 Basic data sheet.
